# Successful experience of tofacitinib treatment in patients with Fibrodysplasia Ossificans Progressiva

**DOI:** 10.1186/s12969-023-00856-1

**Published:** 2023-08-29

**Authors:** Irina P. Nikishina, Svetlana V. Arsenyeva, Valeria G. Matkava, Alia N. Arefieva, Mariya I. Kaleda, Alexandr V. Smirnov, Leonid M. Blank, Mikhail M. Kostik

**Affiliations:** 1 V.A. Nasonova Research Institute of Rheumatology, Kashirskoe shosse, 34A, Moscow, 115522 Russia; 2 Hospital Pediatry Department of Saint-Petersburg State Pediatric Medical University, Saint-Petersburg, Russia

**Keywords:** Fibrodysplasia ossificans progressiva, FOP, Heterotopic ossification, Tofacitinib, Autoinflammation, Spondyloarthritis-like disease, *ACVR1* gene, Bone morphogenetic protein, BMP

## Abstract

Fibrodysplasia ossificans progressive (FOP) is an ultra-rare genetic disorder that is caused by a mutation in the *ACVR1* gene and provokes severe heterotopic ossification. Since flares of the disease are associated with inflammation, it is assumed that JAK inhibitors can control active FOP due to blocking multiple signaling pathways.

## Abstract (summary)

**Aim of Study:**

To assess the safety and efficacy of tofacitinib in patients with FOP refractory to standard of care treatment.

**Material and Methods:**

In the retrospective observation study we included information about 13 genetically confirmed FOP patients who were treated with tofacitinib 5 mg twice a day. All patients initially failed treatment with NSAIDs, corticosteroids, and bisphosphonates. We evaluated the patients 12 months before the beginning of their treatment with tofacitinib and continued evaluating to at least 12 months of their tofacitinib treatment period.

**Results:**

In 7 boys (54%) and six girls (46%) tofacitinib was prescribed at the age of 10.2 (ranged 2.2–19.6) years. Onset age was 1.5 (ranged 0.1-6.0) years. Main clinical features were malformed great toes (n = 13; 100%), short malformed thumbs (n = 9; 69%), peripheral osteochondromas (n = 9; 69%), abnormalities of the cervical spine (n = 13; 100%), multiple heterotopic ossifications (n = 13; 100%). The mean diagnostics delay was 44 (range: 1; 165) months. During the trial, the median (25%; 75%) frequency of flares decreased from 10 (6; 12) during 12 months before the baseline to 0 (0; 2) in the following 12 months and 0 (0; 0) in 24 months of treatment. There were no deteriorations of the CAJIS index during the study, except for only one patient whose CAJIS index deteriorated by 1 point. Improvement in the range of motion in the large joints was noted in 31% of patients. NSAID, oral and intravenous corticosteroids were successfully decreased from 100%; 61.5%, and 15.4% (baseline) to 46.2%, 7.7%, and 0% (12 months) and 22.2%, 0%, and 0% (24 months), consequently. The drug tolerance was good. No severe adverse events were registered.

**Conclusion:**

Tofacitinib is a highly efficient, well-tolerated option that may prevent FOP flares. Further studies of the therapeutic potential of JAK-kinase inhibitors in FOP patients are needed.

## Introduction

Fibrodysplasia ossificans progressiva (FOP) is an ultra-rare metabolic disease caused by *ACVR1* (Activin A receptor type I ) gene variants with frequency 1:1 400 000–2 000 000. It is one of the most disabling conditions characterized by repeated inflammatory-mediated flare-ups with heterotopic ossification, named a «second skeleton disease» due to uncontrolled osteogenesis [1]. Several cytokines, operating in both bone metabolism and osteogenesis are involved in the FOP pathogenesis, throw NF-κB, BMP pathway hyperactivation [2, 3]. Inflammatory nature and overlapping with rheumatic conditions, e.g. spondyloarthritis it is suspected FOP is an immune-mediated or autoinflammatory disease [4]. Pignolo R.J. and colleagues found that adiponectin which is implicated in hypoxia, inflammation, and heterotopic ossification as well as tenascin-C (an endogenous activator of innate immune signaling through the TLR4 pathway and a substrate for kallikrein-7) were highly correlated with FOP genotype, while kallikrein-7 was highly correlated with acute flare-up status in 40 FOP patients with typical ACVR gene variant [5]. Barruet E. with colleagues described increasing levels of proinflammatory (IL-3, IL-7, IL-8) and anti-inflammatory IL-10 cytokines in FOP [2]. Corticosteroids decrease inflammation and prevent new bone formation, especially in the injured tissues. Treating with anti-IL-1 agents (anakinra switching to canakinumab) decreased the rate of FOP flares and/or limit the symptoms and residual lesions. The studied cytokine profile (high levels of IL-1 β up to 21.5 pg/ml were detected) and efficacy of anti-IL-1 treatment shed light on FOP immunopathogenesis and allowed us to consider the FOP as an autoinflammatory disease [6]. According to our previous study high level of IL-1RA and TNFR2 was detected in patients with FOP [7]. To date, there are no drugs with fully proven efficacy of completely suppressing the ossifications in FOP. Several clinical trials of new promising drugs are ongoing. Only the RARγ-Specific Agonist palovarotene study has been completed, and the results have not been fully published yet. Food and Drug Administration (FDA) and European Medicines Agency (EMA) have not approved yet the use of palovarotene in FOP patients. Corticosteroids and non-steroid anti-inflammatory drugs (NSAID) might be effective in the treatment of disease flares confirming the inflammatory basis of the disease [1]. This knowledge of the pathogenesis of the disease makes it possible to consider other biologic and non-biologic disease-modifying antirheumatic drugs (DMARDs) as the possible effective treatment options for FOP, but the injection route of administration may provoke the FOP flares. Janus kinase (JAK) inhibitors are the new class of synthetic DMARDs has a comparable efficacy with biologic DMARDs, but have an oral route of administration, which is preferred for patients with FOP. Tofacitinib recently was approved by FDA for polyarticular juvenile idiopathic arthritis (JIA) [8]. We suggested that the anti-inflammatory effect of tofacitinib can control the flares of FOP and prevent or reduce the ossifications. Our study aimed to assess the safety and efficacy of tofacitinib in patients with FOP refractory to standard of care treatment.

## Patients and methods

### Patient selection

In the retrospective observational study, we included information from patients’ case report forms about 13 genetically confirmed FOP patients (7 males, 6 females) aged from 2 to 19 years who have been treated with tofacitinib 5 mg twice a day. All patients initially failed treatment with NSAIDs, corticosteroids, and bisphosphonates. Our cohort consists of 44 FOP patients and 20 of them were treated with tofacitinib.

#### Inclusion criteria

(i) *ACVR1* genetic variants accompanied with relevant disease course; (ii) signed informed consent; (iii) inefficacy of previous standard of care treatment, including NSAIDs, corticosteroids, and bisphosphonates; (iv) available clinical information in 12 months before and at least 12 months after tofacitinib administration (v) tofacitinib treatment duration at least 12 months; (vi) inclusion age more than two years; (vii) active phase of disease at baseline with active flares in the previous 12 months.

In each patient, we evaluated 12 months period before tofacitinib administration (baseline) and at least a 12-month tofacitinib treatment period.

### Assessments and outcomes

*During the study, we analyzed the following characteristics*:

- demography: gender, onset age, diagnostics delay.
- clinical characteristics: the presence of malformed great toes, short malformed thumbs, cervical spine abnormalities, peripheral osteochondromas, number of flare-ups. Flare means the new node formation with swelling, local hyperthermia, tenderness, or exacerbation of existing nodes, confirmed clinically.
- Functional disability status was evaluated by the Cumulative Analogue Joint Involvement Scale (CAJIS) [9].
- laboratory data, including *ACVR1* gene variants. Routine blood tests and biochemistry was monitored in all patients.
- blood samples for investigating inflammatory biomarkers were collected during the flare at baseline and routine follow-up visits in the inactive phase of the disease. Serum levels of IL-18, IL1RA, IL1b, IL-6, TNFR1, TNFR2, and ferritin were measured using standard commercial enzyme-linked immunosorbent assay (ELISA). C-reactive protein (CRP) was determined by the commercial nephelometric method.
- imagine data: joint ultrasound (hip, knees, ankles) for assessment of synovitis; X-ray of cervical spine and chest; whole body or regional magnetic-resonance imaging (MRI); Low-dose whole-body computed tomography (WBCT) was performed.

### Outcomes

#### Number of flare-ups

To assess the effectiveness of therapy, we evaluated the number of flare-ups 6, 12, 18, and 24 months after baseline (start of tofacitinib) and compared them with 12 months before baseline.

#### CAJIS

Functional disability status was evaluated by CAJIS in all patients in baseline and 6,12,18,24 months after [6].

We evaluated the part of patients at each time-point (baseline, 6, 12, 18, and 24 months) who had clinical improvement in the range of motion at least in one large joint according to the opinion of the attending physician.

#### Concomitant treatment dynamics

At every time point, we evaluated the number of patients, being treated with NSAID, oral and intravenous corticosteroids since the baseline. In the case of combined therapy (oral corticosteroids + NSAIDs), corticosteroids were tapered first as usual at 1 mg of methylprednisolone per week according to the physician’s opinion and were successfully discontinued. NSAIDs were discontinued during the 1st month after corticosteroids were discontinued. In the case of a flare-up, the duration of NSAID might be longer or NSAID might be restarted according to the physician’s opinion. Intravenous corticosteroids could be used in the pulse-therapy regimen with methylprednisolone 10–20 mg/kg for three consecutive days during the flare-ups in cases if NSAID failed.

**Fig. 1:**
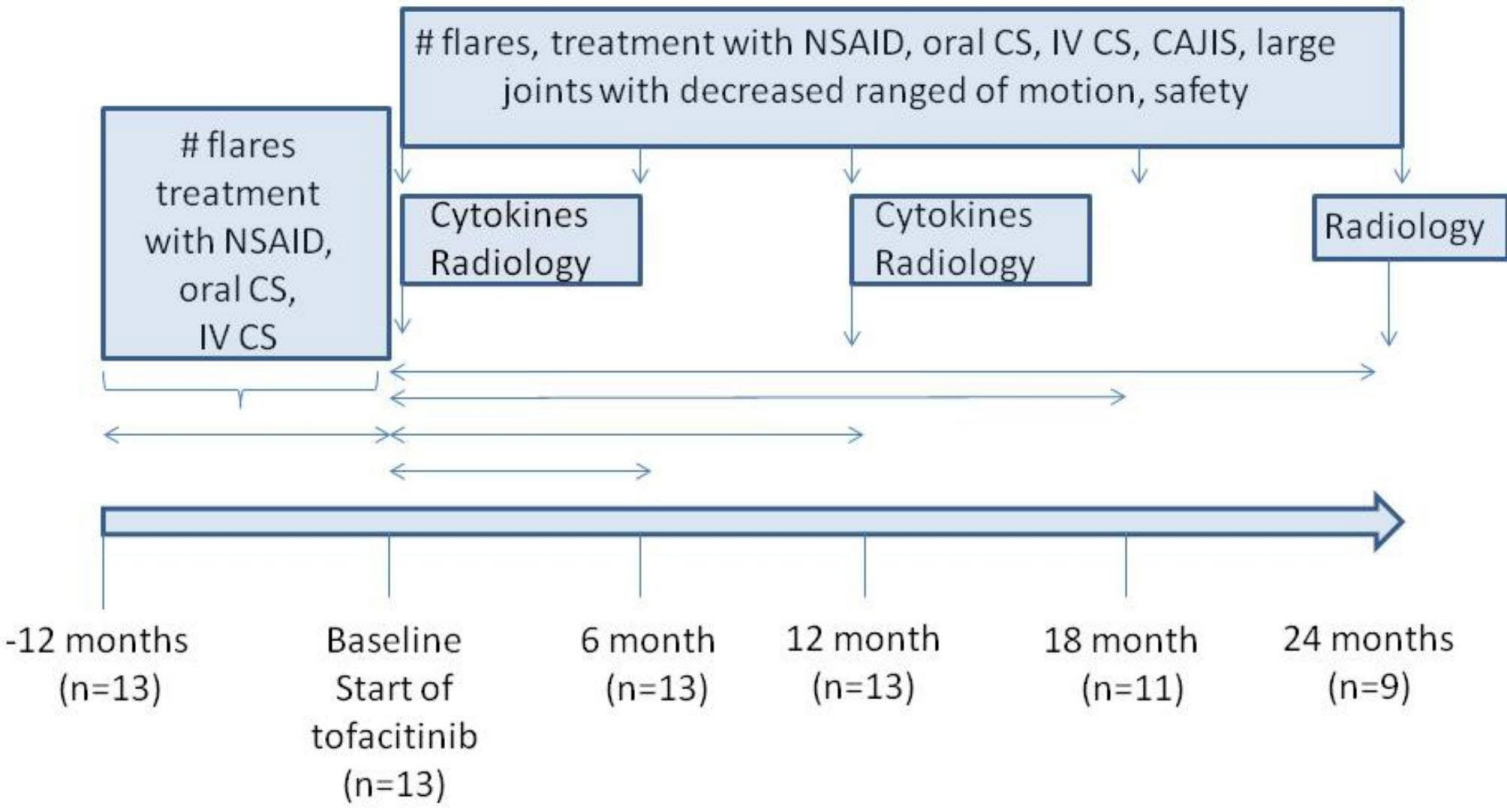
The study flow-chart with timelines

***Safety evaluation*** – the overall frequency of any adverse events, including laboratory abnormalities, and acute infections frequency at 6, 12, 18, and 24 months compared to the 12-month interval before the baseline. The study timeline is in Fig. 1.

### Statistics

Descriptive statistics are reported in terms of medians (Me) and interquartile ranges (IQRs) and means (M) and standard deviations for continuous variables and absolute frequencies and percentages for categorical variables. We used a non-parametric statistic because all variables had non-normal distribution. To check whether the distribution was normal or not, we used the Kolmogorov-Smirnov test. Wilcoxon’s matched pairs test was used to compare two dependent continuous variables, Friedman’s test for more than two continuous variables, and Mac-Nemar’s test for dependent categorical ones. The software Statistica (release 10.0, StatSoft Corporation, Tulsa, OK, USA) was used for data analysis. P values < 0.05 were considered to indicate a significant difference.

## Results

### Patients’ baseline characteristics

All patients have “classic” FOP phenotype with the typical *ACVR1* gene c.617G > A (p.Arg206His) variant in 12 (92%), and the ultra-rare с.983 G > A (p.Gly328Glu) variant in one patient (8%). All patients had the most severe course with rapid progression of ossification and limitation of movements in the large joints. The median baseline CAJIS score was 9.4 (3; 17) points, ranging from 3 to 17. The maximum score was observed predominantly in older patients. Large joints (knees, hips, and ankles predominantly) effusion was detected by MRI and ultrasound in 13 (100%) patients.

Previous and baseline medications included NSAIDs, oral corticosteroids, intravenous corticosteroids, and intravenous and oral bisphosphonates. The data are in Table 1.

**Table 1:** Baseline demographic and clinical characteristics of FOP patients

Characteristic of FOP patients, n = 13	
Sex: boys/girls, n (%)	7 (54) / 6 (46)
Onset age, Me (25%; 75%), years	1.0 (0.5; 2)
‒ Mean (min; max), years	1.5 (0.1; 6.0)
Age of tofacitinib administration, Me (25%; 75%), years	10.4 (4.4;16.5)
‒ Mean (min; max), years	10.2 (2.2;19.6)
The time between disease onset and tofacitinib administration, Me (25%; 75%), years	4.2 (3.4;11.5)
‒ Mean (min; max), years	7.3 (0.75;18.3)
Diagnostics delay, Me (25%; 75%), months	15 (3;73)
‒ Mean (min; max), months	44 (1;165)
**Features of FOP, n = 13**	
Malformed great toes^*^, n (%)	13 (100)
Short malformed thumbs^*^, n (%)	9 (69)
Сervical spine abnormalities^*^, n (%)	13 (100)
Multiple heterotopic ossifications^*^, n (%)	13 (100)
Peripheral osteochondromas^*^, n (%)	9 (69)
CAJIS Me (25%; 75%), score	7 (6; 14)
‒ Mean (min; max), score	9.4 (3;17)
*ACVR1* gene variants:	
‒ typical: c.617 G > A p.Arg206His, n (%)	12 (92)
‒ ultra-rare variant: с.983 G > A (p.Gly328Glu), n (%)	1 (8)
**Imaging**	
Bilateral sacroiliitis	6/7 (86%)
Bilateral hip joints effusion:	
Ultrasound investigation	13/13
Magnetic resonance imaging	7/7
Previous treatment experience n (%)	
Nonsteroid anti-inflammatory drugs	13 (100)
Corticosteroids, oral	13 (100)
Corticosteroids, intravenous	8 (62)
Bisphosphonates	8 (62)
Baseline treatment experience, n (%)	
Nonsteroid anti-inflammatory drugs	13 (100)
Corticosteroids, oral	8 (62)
Corticosteroids, intravenous	2 (15)

**Abbreviations** FOP - fibrodysplasia ossificans progressive; CAJIS - Cumulative Analogue Joint Involvement Scale; ^*^calculated per patient

### Tofacitinib treatment efficacy

The duration of tofacitinib therapy ranges from 12 to 36 months (Me = 24.2 months). Thirteen patients were treated with tofacitinib at 12 months, 11 patients - at 18 months, and 9 patients – at 24 months.

#### Dynamics of flare-ups

**Fig. 2:**
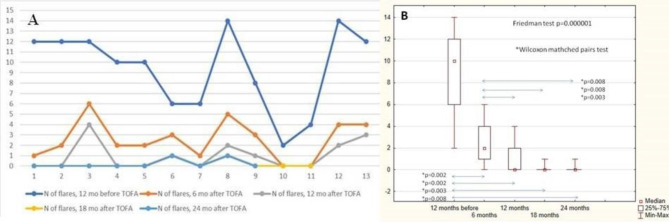
A) The dynamics of flare numbers during the study **(A)** By patient; **(B)** By time points

The median number of flares 12 months before tofacitinib treatment was 10 (6; 12), range: 2–14. After the first 12 months of tofacitinib therapy, the number of new flares significantly decreased. The median number of flares 6 months after tofacitinib treatment was 2 (1; 4), range: 0–6. The median number of flares 12 months after tofacitinib treatment was 0 (0; 2), range: 0–4. In one patient after 6 months of tofacitinib therapy, the treatment was temporally stopped due to lack of treatment access and it led to new flare-ups and a right elbow joint block. The median number of flares 18 months after tofacitinib treatment (n = 11) was 0 (0; 1), range: 0–1. The median number of flares 24 months after tofacitinib treatment (n = 9) was 0 (0; 0), range: 0–1. The data are in Supplementary Table, S1 and Fig. 2.

### CAJIS dynamics

The CAJIS score has been stable since baseline in 12 patients. CAJIS index deteriorated by one point in one patient.

### Large joint’s range of motion dynamics

In four patients we observed improvement in the range of motion in the large joints especially in the shoulders since the baseline.

### Biomarkers

We analyzed repeatedly the spectrum of several serum biomarkers in a small group of patients with FOP (n = 5) in paired serums before and under the tofacitinib treatment. During the trial, the levels of IL1RA decreased in 4/5 (80%) and increased in 1/5 (20%). The levels of IL18 decreased in 1/5 (20%), increased in 1/5 (20%), and were unchangeable in 3/5 (60%). The levels of IL10 increased in 3/5 (60%), decreased in 1/5 (20%), and were stable in 1/5 (20%). The levels of IL-6 decreased by 3/5 (60%) and increased by 2/5 (40%).

### Concomitant treatment dynamics

**Fig. 3:**
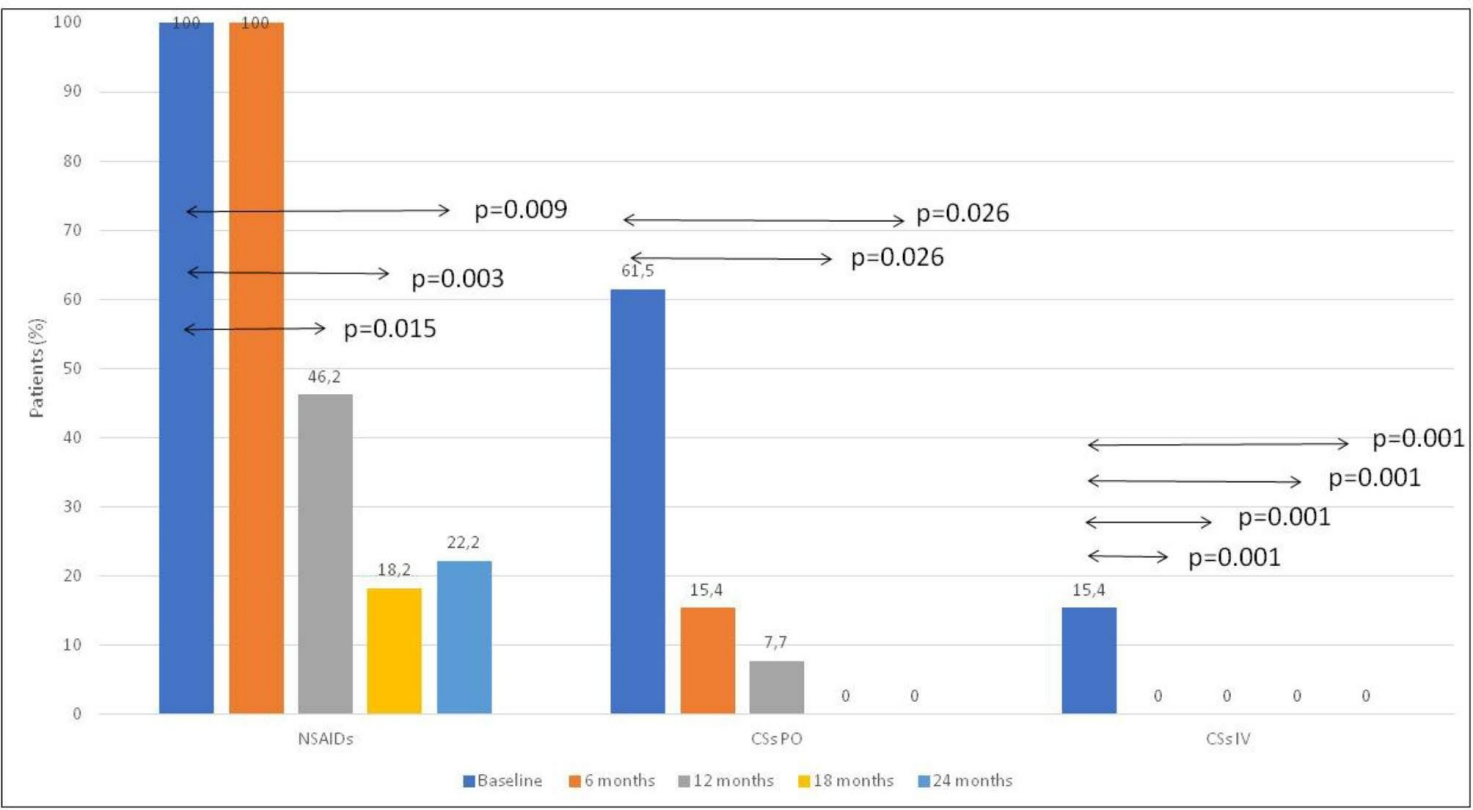
The dynamics of concomitant therapy during the study. **Abbreviations**: NSAID – the part of patients, treated with non-steroid anti-inflammatory drugs; CSs PO – the number of patients treated with oral corticosteroids; CSs IV - the number of patients treated with intravenous corticosteroids

Corticosteroids were completely discontinued in 6/8 (75%) in the first six months of the study. The remaining two patients discontinued them after 8 (11 years old) and 14 months (2 years old) since the baseline. There was no new or repeated administration of corticosteroids during the follow-up. Every new flare was effectively treated with only short-term courses of NSAIDs (2–3 weeks) without corticosteroids. Data are in the Supplementary Table, S1 and Fig. 3.

### Drug safety

The drug tolerance was good in all patients, no severe adverse events were registered. The rate of acute infections during and before the trial was not changed. No laboratory abnormalities, no cytopenia, required temporary or permanent discontinuation of tofacitinib were observed, as well as thrombotic events. Two patients had the new SARS-CoV-2 infection in the mild form.

### Imaging

**Fig. 4:**
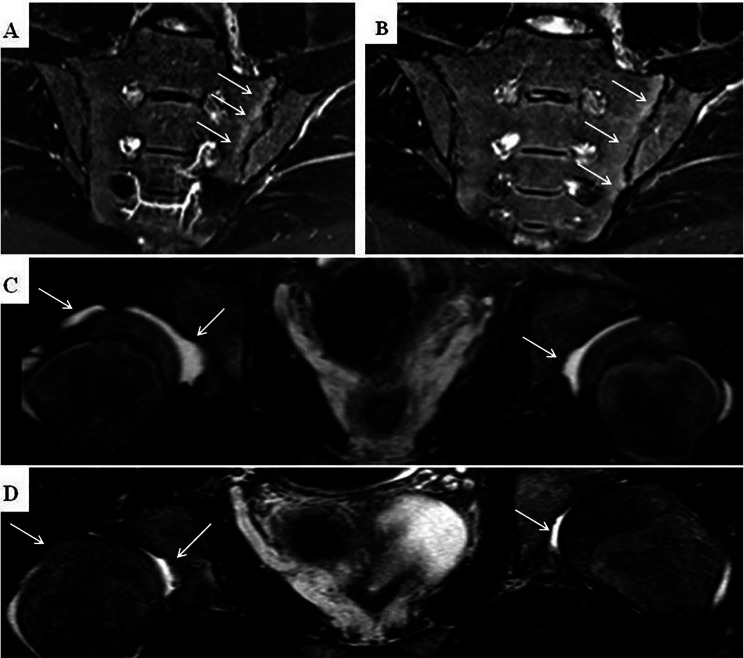
MRI of hips and sacroiliac joints of FOP patients during the study. **A** – active sacroiliitis in 18 years old male patient before tofacitinib therapy (the subchondral bone marrow edema noted by the arrows). **B** – the same patient after 1 year of tofacitinib treatment (reduction of the subchondral bone marrow edema, arrows). **C** – MRI evidence of bilateral hips effusion in 12 years old male patient before tofacitinib therapy (arrows). **D** – the same patient 1.5 years after tofacitinib treatment, regression of synovial effusion in the hip joints (arrows)

The signs of bilateral sacroiliac joint involvement were detected in 6/8 (75%) of FOP patients: in 5/7 cases with MRI and in 1/1 with CT and X-ray due to the impossibility to perform MRI (severe skeletal deformities). Low-dose whole-body CT (WBCT) re-confirmed sacroiliitis in two patients with MR-positive signs of sacroiliac joint involvement. Repeated MRI demonstrated regression of active sacroiliitis in 4/5 (80%) patients and hip joint effusion in 7 (100%) patients. The examples of imaging dynamics are in Fig. 4.

## Discussion

In our study, the safety and efficacy of tofacitinib in active FOP were demonstrated. Tofacitinib decreases the number of flares, improves the range of motion in large joints and imaging characteristics, and allowed to decrease in the concomitant medications, especially systemic corticosteroids in active FOP patients, which fell to previous treatment. The number of flares progressively decreased according to the tofacitinib treatment duration. The treatment efficacy depended on the patient’s age. Interestingly, the younger patients (from 2 to 4 years old) had longer persistent disease activity with the formation of new inflammatory lumps possible due to their increased physical activity, traumatization (falls), and more frequent infections.

The FOP treatment consists of two approaches. Firstly, the treatment of new flares/foci with short-term corticosteroids and NSAIDS, and secondly, the basic treatment prevented new flares/new foci formation/ new bone volume progression. International guidelines recommend a short four-day course of high-dose corticosteroids (including an intravenous regimen) within the first 24 h of a flare-up or 3–4 days of oral prednisone (1–2 mg/kg) days after severe soft-tissue injury [10]. It is well known, that FOP flares are driven by inflammation, and the efficacy of early corticosteroid administration in the acute edema phase (before new bone formation) confirms this theory, as well as temporarily increased acute phase reactants and pro-inflammatory cytokine during the flares. Disease-modifying FOP treatment, preventing new flares and new bone formation is a challenging problem. A long course of corticosteroids, chronic NSAID treatment, and bisphosphonates have been recommended as a disease-modifying treatment for the last decades. Corticosteroid dependence and toxicity require limiting the use of these drugs as the main long-term treatment option. Bisphosphonates, used in FOP treatment block osteoclasts, and provide an anti-inflammatory effect, which was confirmed in patients with chronic multifocal osteomyelitis and ankylosing spondylitis [11]. It is unclear, which of the two effects works better in FOP patients. The blockade of osteoclasts should stop the bone remodeling cycle and theoretically should decrease the source of calcium for new bone formation. Surviving osteoblasts, accompanied by osteoclast dysfunction leads to increased bone mineral density during bisphosphonate treatment. It is also unclear, shall we consider this effect beneficial for FOP patients or not. The main FOP treatment strategy is a repositioning of some previously approved drugs for other indications, such as mast cells inhibitors or tyrosine kinase inhibitor – imatinib, which was originally indicated for patients with chronic myeloid leukemia [12, 13]. One more new approach is a potent inhibitor of the protein kinase ALK2 saracatinib is investigated in the STOPFOP study [14]. Some data suggest that saracatinib is an efficacious clinical candidate for repositioning for FOP treatment, offering an accelerated path to clinical proof-of-efficacy studies and potentially significant benefits to individuals with this devastating condition [15]. The effect of protein kinase ALK2 and tyrosine kinase inhibitors are similar to tofacitinib and related to multiple cytokine inhibition throw JAK/STAT signaling pathway, but tofacitinib is more convenient for chronic treatment being a typical anti-rheumatic drug with better tolerance and safety profile, confirmed by the several clinical trials and real-world data of adult patients [16]. The pediatric experience is growing now since the drug was approved for juvenile idiopathic arthritis in 2021 and is not limited to the data from the third phase of a double-blind, placebo-controlled trial in polyarticular juvenile idiopathic arthritis [8].

Real-world data showed the efficacy of tofacitinib in high-disease activity patients with resistance to corticosteroids and/or previous biologics treatment in patients with JIA (n = 15), systemic autoinflammatory diseases (n = 7), juvenile dermatomyositis (n = 2) [17]. According to our experience with tofacitinib in additional 52 pediatric patients with different pediatric rheumatic diseases, including a resistant form of JIA and rare autoinflammatory diseases, such as Blau syndrome, CANDLE, SAVI, CACP, tofacitinib seems to be a promising option of treatment with different immune-mediated disease, not limited to JIA [18].

The first pathogenetic drug for FOP treatment is RARγ-Specific Agonist – palovarotene. A placebo-controlled, double-blind trial was completed recently. Palovarotene diminished the proportion of patients with FOP, experienced new flares, and decreased the volume of new bone after flare-ups, compared with placebo [19]. The new treatment options for FOP patients are pending. Nowadays several clinical trials have been investigating some new molecules with a different mechanism of action such as garetosmab - a fully human monoclonal antibody that inhibits activin A [20].

The experience of rheumatologists and the repositioning of some anti-inflammatory targeted drugs for the treatment of active manifestations of FOP are promising. The mechanism of JAK-inhibition seems to be preferable compared to the direct anti-cytokine effect of most biologics due to the multi-targeting inhibition of the inflammation in the FOP. The oral route of administration avoiding tissue injuries, which may provoke the disease flare makes additional benefits than injected mono-targeting biologics. Achievement of steroid-fee remission is one of the important tofacitinib treatment outcomes.

The study limitation is related to a very small sample size of the studied population, the absence of the control group, and the lack of imaging evidence of new bone volume dynamics.

## Conclusion

Tofacitinib is a highly efficient, well-tolerated treatment option for a severe persistent course of FOP for the prevention of new flares and achieving control over the activity of the disease. The oral route of administration and the possibility to escape any injections in FOP patients are extremely important. Further studies of the therapeutic potential of JAK-kinase inhibitors in FOP patients are needed.

## Data Availability

The original contributions presented in the study are included in the article/supplementary material, further inquiries can be directed to the corresponding author.

## List of Abbreviations

ACVR1: Activin A receptor type I
CACP: Camptodactyly, Arthropathy, Coxa vara, Pericarditis
CAJIS: Cumulative Analogue Joint Involvement Scale
CANDLE: Chronic atypical neutrophilic dermatosis with lipodystrophy and elevated temperature
CAPS: Cryopyrin-associated periodic syndromes
CRP: C-reactive protein
CS: Corticosteroids
CT: Computed tomography
DMARDs: Disease-modifying antirheumatic drugs
EMA: European Medicines Agency
FDA: Food and Drug Administration
FOP: Fibrodysplasia ossificans progressive
JAK: Janus kinase
JIA: Juvenile idiopathic arthritis
IL: Interleukin
MRI: Magnetic-resonance imaging
NSAID: Nonsteroid anti-inflammatory drugs
SARS-CoV-2: Severe acute respiratory syndrome-related coronavirus 2
SAVI: STING-associated vasculopathy with onset in infancy
TF: Tofacitinib
WBCT: Low-dose whole-body computed tomography
